# Evaluation of Patterns of Presentation, Practice, and Outcomes of Upper Tract Urothelial Cancer: Protocol for an Observational, International, Multicenter, Cohort Study by the Clinical Research Office of the Endourology Society

**DOI:** 10.2196/15363

**Published:** 2020-01-24

**Authors:** Joyce Baard, Merve Celebi, Jean de la Rosette, Antonio Alcaraz, Shahrokh Shariat, Luigi Cormio, Vítor Cavadas, M Pilar Laguna

**Affiliations:** 1 Amsterdam UMC University of Amsterdam Amsterdam Netherlands; 2 Istanbul Medipol University Istanbul Turkey; 3 Hospital Clinic i Provincial de Barcelona Barcelona Spain; 4 Medical University of Vienna Vienna Austria; 5 University of Texas Southwestern Dallas, TX United States; 6 Motol Hospital Charles University Prague Czech Republic; 7 IM Sechenov University Moscow Russian Federation; 8 University of Foggia Foggia Italy; 9 Centro Hospitalar Universitário do Porto Porto Portugal

**Keywords:** upper urinary tract, urothelial cancer, incidence, management, outcomes, registry

## Abstract

**Background:**

Available guidelines on the management of upper tract urothelial carcinoma (UTUC) are restricted due to the lack of strong evidence–based recommendations. Adequate, well-powered randomized trials are missing due to the rarity of the disease. To overcome this problem, we need alternative study designs to provide generalizable data.

**Objective:**

The primary aim of this registry is to provide a real-world overview on patterns of presentation and management of UTUC. Secondary objectives include comparison of outcomes of different treatments and tumor stages and evaluation of compliance with the current European Association of Urology recommendations for UTUC.

**Methods:**

For this observational, international, multicenter, cohort study, clinical data of consecutive patients suspected of having UTUC, irrespective of type of management, will be prospectively collected up to 5 years after inclusion. Data on the patterns of presentation, diagnostics, and treatment as well as short-, mid-, and long-term oncological and functional outcomes will be analyzed. Possible associations between variables, basal characteristics, and outcomes will be tested by multivariable analyses. The methodology will address potential sources of bias and confounders.

**Results:**

The registry was initiated in November 2014 after obtaining institutional review board approval. Data collection started in December 2014. At the time of submission of this manuscript, 2451 patients from 125 centers from 37 countries were included. Inclusion of patients will be closed 5 years after initiation of the registry. Quality checks will be performed centrally with continuous communication and feedback with the centers to ensure accuracy. The first results are expected in the first trimester of 2020.

**Conclusions:**

This large observational prospective cohort will generate landmark “real-world” data and hypotheses for further studies. We expect these data to optimize the management of UTUC, provide insights on harms and benefits of treatment, and serve as quality control.

**Trial Registration:**

ClinicalTrials.gov NCT02281188; https://clinicaltrials.gov/ct2/show/NCT02281188.

**International Registered Report Identifier (IRRID):**

DERR1-10.2196/15363

## Introduction

### Background

Upper tract urothelial carcinomas (UTUCs) have a low prevalence, with an estimated annual incidence of 2 per 100,000 inhabitants in Western countries [1]. Most of them are urothelial cell carcinomas arising from the lining endoluminal urothelium, and they represent 5%-10% of all urothelial carcinomas [1]. At diagnosis, nearly 60% of UTUCs are found to be invasive, a much higher percentage than the 15%-20% reported for its sibling tumor in the bladder [1,2]. Although the general genotype of UTUC is similar to its bladder counterpart, it is different in its genetic expression and frequency by stage [3-5]. UTUC may present in a primary isolated form in the upper urinary tract or secondary form after a primary diagnosis of bladder urothelial carcinoma. Synchronous upper and lower urinary tract urothelial carcinoma has been reported in up to 17% of cases [6].

Irrespective of whether it is because of advanced diagnostic methods utilization, decreased competing death causes, or real increasing incidence, the rate of invasive UTUC tumors (>pT1) at diagnosis has been increasing in recent years [1,6]. Metastases are found in 7% of all patients with UTUCs. The 5-year survival does not reach 50% for patients with pT2/pT3 UTUCs and is lower than 10% for pT4 tumors [7,8].

The European Association of Urology (EAU) guidelines recommend risk stratification of UTUC in low- and high-risk disease, based on clinical and pathological factors. These include focality and size of the tumor, presence of high-grade urinary cytology or high-grade histology on biopsy, imaging characteristics, and presence of previous radical cystectomy for bladder cancer or variant histology [1,7]. This risk classification is intended to drive treatment, and some of these risk factors, either isolated or integrated, have prognostic implications [7-9].

The gold standard of UTUC remains radical nephroureterectomy (RNU) with complete bladder cuff excision [10,11]. However, there is a growing interest in minimally invasive, kidney-sparing approaches by ureterorenoscopy (URS). This shift in treatment management is fueled by the development and evolution of flexible instruments. Indications for kidney-sparing management are discussed in detail in the current guidelines and should be considered in patients with low-risk disease [1,7,12]. A particular challenge during endoscopic management is appropriate grading and staging of tumors, both necessary for accurate risk assessment, which is essential for successful therapeutic management [1,13].

Rates of recurrence, either distant or local, are correlated with various factors, the most important of which are pathological stage and grade. A common site of recurrence after treatment of UTUC is the bladder. Intravesical recurrence following RNU is a common problem, with an incidence of nearly 20%-50% [1]. In a meta-analysis (4057 participants) assessing the impact of diagnostic URS prior to RNU, a strong correlation was found between previous URS and development of intravesical recurrence during follow-up after RNU (hazard ratio=1.53, *P*<.001) [10]. Tumor location also plays a role in the choice of therapy based on anatomic location [1,6,14].

### Rationale for a Clinical Registry

Available clinical information on the management of UTUC relies mainly on historical cohorts. Monocentric data from merging international databases or population-based studies have provided the highest level of evidence so far and are challenging the standard management algorithms [15-17]. Although valuable, this type of information is prone to bias. Patient selection criteria, attrition bias, and verification bias are among the most frequent confounders in spite of efforts to adjust for them. Even in large cohorts, confounding variables cannot be completely corrected for. Although associations may be observed, they rarely confirm causality and may have unknown effects [18,19].

In an intent to increase the quality of evidence, systematic reviews and meta-analysis have explored specific UTUC outcomes of distinct treatment modalities as well as predictive and prognostic factors [19-27]. Obviously, they provide insights in the natural history of UTUC, but they are still the product of a low-evidence report. Therefore, they do not result in strong recommendations due to the flaws in accuracy and generalizability [28].

Although randomized trials (RCTs) would provide sound answers, the rarity of the condition prevents studies from obtaining an adequately powered sample size for correct comparison in a reasonable study time. The late onset and comorbidity of the affected population will limit inclusion and likely preclude generalizability of RCT results. In this scenario, it is rational to rely on alternative study designs that allow rapid data collection with inclusion of a large population with a broader geographic and ethnic spectrum [29,30].

Clinical registries are defined as “a system that collects a defined minimum data set from patients undergoing a particular procedure, diagnosed with a disease or using the health care resource” [29]. They are observational databases focusing on a specific clinical condition, therapy, or population without specific mandate approaches and are intended to reflect “real world” practice in a large population. When properly designed and executed, they serve to improve the quality of health care and as hypothesis generators [29].

Very little is known about the prevalence of registries worldwide. Overall, the interest has increased rapidly in the last decade in countries such as the United States, Sweden, and the United Kingdom, with more than 100 registries per country. Whether named registries, quality registries, clinical databases, clinical audits, or quality improvement programs, the medical societies unanimously recognized their value in the clinical research context [31,32].

We hereby present the design of the Clinical Research Office of the Endourology Society (CROES)-UTUC registry.

### Objectives

Overall, the registry aims to provide a contemporary real-world overview and generalizable comparative outcomes on the incidence and management of UTUC across the globe. The CROES-UTUC registry focusses primarily on incidence, indications, treatment, and patient outcomes. When possible, comparative clinical effectiveness of different interventions and assessment of safety of these interventions will be performed.

The primary objective is to describe contemporary patterns of presentation, practice, and treatment of UTUC according to geographic characteristics.

The secondary objectives identified by the steering committee during the design of the registry include several short- and long-term comparative outcomes listed below:

- To assess the compliance with the current EAU guidelines on UTUC recommendations
- To assess the validity of risk stratification, as recommended by the current EAU guidelines on UTUC
- To assess the intra- and postoperative complications stratified by type of treatment
- To determine the rates and type of recurrences (upper tract and bladder/local or distant) as well as risk factors for patients presenting with primary UTUC stratified by type of treatment (kidney-sparing treatment vs radical nephroureterectomy vs segmental ureterectomy), stage at presentation, select clinicopathologic characteristics, and gender
- To determine comparative overall survival stratified by type of treatment and stage at presentation.

## Methods

### Study Design

This is an observational, international, multicenter, cohort study, prospectively collecting clinical data on consecutive patients with UTUC. The registry is set up by the CROES, and its design follows the recommendations of the Agency for Healthcare Research and Quality (US) of 2014 for design and use of patient registries for scientific, clinical, and health policy purposes [29].

**Figure 1:**
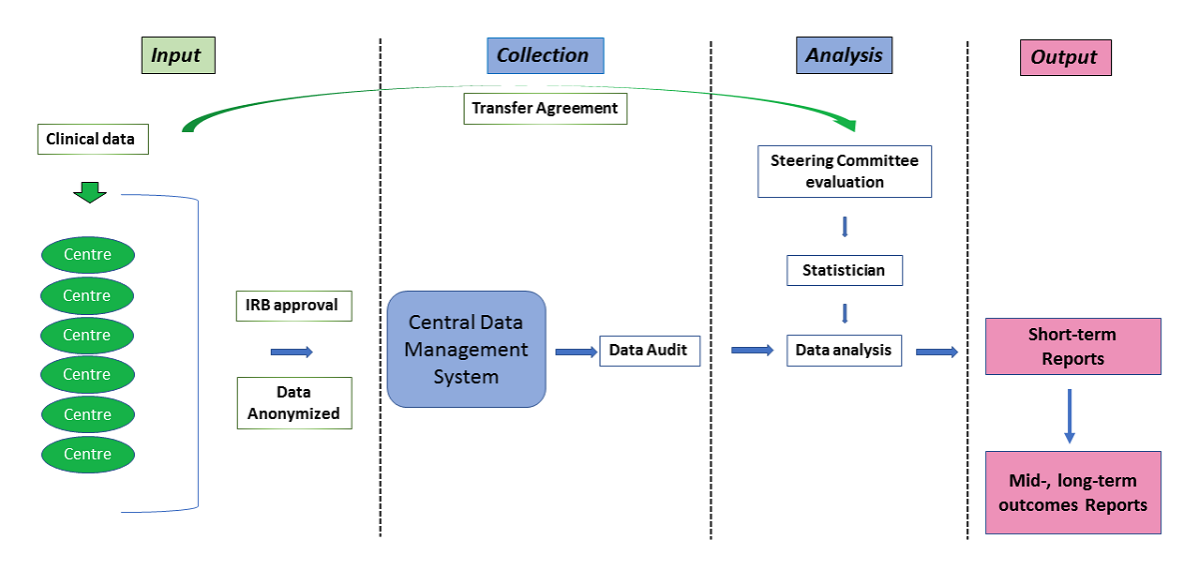
Operational flow diagram of the CROES-UTUC (Clinical Research Office of the Endourology Society for urothelial carcinomas of the upper tract) registry. Colors indicate the different levels of data input, management, and output as well as the interaction directions among them. Green color indicates external sources of information (participating centres); blue colour indicates central registry office tasks; pink/blue color indicates a combination of centres and central office registry output. IRB: institutional review board.

This registry collects clinical data on patients with UTUC, irrespective of onset, location, or type of treatment. It enrolls patients at the moment of their health care visits and includes baseline information on demography, symptoms, risk factors, and laboratory variables. Diagnostic procedures are captured as well as management, treatment, and follow-up details (up to 5 years after inclusion). In summary, it captures patterns of presentation, diagnostics, and treatment as well as short-, mid-, and long-term oncological and functional outcomes. The operational flow diagram design of the registry is provided in Figure 1. Patients were not involved in the design of the registry.

Version 2 of the protocol was registered in September 2014 in Clinical trials.gov (trial registration: NCT02281188). The data and material for this study are available from CROES upon request.

A steering committee composed of six international experts in the field oversaw the design process and checked the structure of the registry. Primary and secondary objectives were defined before design and initiation of the registry. The different centers have the opportunity to identify “ad hoc” secondary objectives and propose studies across the duration of the study. The steering committee will review the different study proposals; modify the proposal, if necessary; and consequently approve or deny them. Proposals will be handled according to chronological submission.

Due to the amount of data and the opportunity for the centers to propose studies, not all secondary objectives were predefined prior to initiation of the registry. They will be identified “ad hoc” by the participating centers or the steering committee according to the data available at the time of study proposal. We foresee that the large sample size will allow for comparative studies on benefit of interventions, evaluation and definition of risk factors for recurrence, as well as associations and quasi-randomized outcomes comparisons.

### Participant Characteristics

Adult patients (age≥18 years) suspected of having UTUC either as primary onset or after previous bladder urothelial cancer (during follow-up) and scheduled to undergo any type of diagnostic instrumentation of the upper urinary tract or any surgical treatment (ie, RNU, kidney sparing surgery by URS or percutaneous treatment or segmental ureter or pelvic resection with or without any other neoadjuvant, perioperative, or adjuvant interventions) will be included in the registry. In line with the design and objectives of a registry, the inclusion criteria are broad, while the exclusion criteria are minimized.

No direct benefits or risk for patients are derived from the participation in the registry. The registry data do not imply any change in management policy or practice apart from the standard practice in the respective centers at any moment of the diagnostic and therapeutic follow-up processes.

### Data Collection and Analysis

Data from all participating centers will be collected through electronic case report forms by using an online data management system (DMS). The DMS is a Web-based system, which makes it convenient for participants worldwide to use, and multiple users of the same institution can be connected to the same data. The DMS is located and maintained at the CROES Office. A more detailed overview of the CROES DMS is provided in Multimedia Appendix 1 [33,34].

Local sites will fill out the electronic case report forms in the DMS prospectively and continuously over time and at the appropriate time. The correspondent local principal investigator is responsible for the reliability of the data and for controlling the accuracy of data. Patient data entered in the DMS was coded, and according to the European General Data Protection Regulation that went "into force" in May 2018, that data is pseudonymized. This means that only the controller (principal investigator responsible for the respective center) can link the code to the patient for audit purposes and follow-up data provision. The identity of the patients is not accessible through the CROES-DMS.

The DMS provides detailed overview reports of included data and runs queries to check for data inconsistencies and outlying values in order to ensure a reliable high-quality dataset. To minimize missing data, the CROES Office sends updates of the database to the principal investigator and is responsible for sending reminders to encourage provision of missing or follow-up data. The managers of the CROES office are in charge of monitoring the registry. Reports are regularly made to the steering committee. Data inconsistencies will be addressed by the local principal investigator, and decisions will be made by the steering committee on a case-by-case scenario.

Essential data elements aim to capture the multiple dimensions of the condition, from diagnosis to survival outcomes; baseline characteristics; risk factors; imaging and clinical assessment; management; complications up to 30 days after intervention; and survival data at 1, 3, and 5 years.

Data variables included in the electronic case report forms are categorized into six domains: general data, pretreatment assessment, treatment, pathology results, postoperative course, and follow-up. Each domain includes multiple variables.

All variables are defined, and they can be categorical (including descriptive, eg, type of complication for rare complications) or continuous. The variables collected include demographic and clinical patient characteristics, risk factors and symptoms, imaging and laboratory tests, treatment type, and pathological and survival outcomes. The key variables are described in Textbox 1.

**Domains, key variables, and total number of variables in the UTUC-CROES registry.**

- General data (number of variables=71)
  - Patient demographics
  - Medical and family history
  - Risk factors
  - Symptoms
  - Medication (anticoagulants)
- Pretreatment assessment (number of variables=80)
  - Imaging (local, regional, and distant)
  - Cystoscopy
  - Cytology
  - Clinical tumor, node, and metastasis stage
- Treatment data (number of variables=141)
  - Use of antibiotic prophylaxis
  - Treatment type
  - Intraoperative details
  - Instrumentation details
  - Neoadjuvant treatment
- Pathological data (number of variables=44)
  - Grade and Stage (2016 World Health Organization International Society of Urologic Pathologists)
  - Pathological Stage
- Perioperative data (number of variables=29)
  - Use of antibiotics
  - Complications up to 30 days (number and type)
  - Clavien-Dindo classification complications
  - Use of intravesical instillation
  - Indication for adjuvant therapy
- Follow-up (number of variables=76; 76 variables per follow-up domain (maximum 5-year follow-up). Maximum of 21 follow-up visits possible in the data management system per patient.
  - Survival status
  - Presence of recurrence
  - Location of recurrence
  - Diagnostics performed
  - Results of the diagnostics
- Total variables (number of variables=441)

As described in the operational flow diagram of the registry, the CROES statisticians will perform statistical analysis after data audit and data cleaning. The analysis will be performed after identification of specific objectives and attaining study approval from the steering committee.

Descriptive statistics will be used to summarize the data. Results will be presented in tables reporting at least the number of subjects, mean, SD, and minimum and maximum for continuous data and the number of subjects and percentages for categorical data. For testing, a significance level of 5% will be maintained, and all tests will be two-sided.

All analyses will be carried out on available data, and proportions of missing data will be reported. All analyses will be performed using SPSS version 25 (IBM Corporation, Armonk, New York) or R studio (RStudio Team, Boston, Massachusetts).

Multivariable analyses will be performed to assess possible associations with geographical or ethnic differences and risk factors for complications after surgery. The methodology will address potential sources of bias. When necessary, sensitivity analysis will be conducted. We will not use external data sources for comparison unless they are considered to be of outmost importance for specific objectives.

After closure of the primary inclusion process, an audit will be planned. The audit will focus on data source verification of the values of the identified critical variables and on internal consistency by cross checking among exclusive variables.

### Availability of Data

Individual centers have signed a data transfer agreement. The centers are responsible for providing data and can use their own data for individual publications upon request and authorization of the steering committee of the registry.

The steering committee will revise and give final approval to any paper derived from the data collected in the course of the study and will determine authorship based on contribution on any paper derived from this registry. Findings and reports derived from this registry will be presented at international urology and oncology conferences and published in peer-reviewed journals. CROES will summarize the findings on the Web by regular information letters [35].

## Results

The registry was initiated in November 2014 and aims to recruit up to 3000 patients in a 5-year period. The study has been registered at the competent authority for observational studies. Institutional review board approval was requested and judged not necessary according to the Medical Research Involving Human Subjects Act (date of resolution: October 15, 2014; ref W14-273#14.17.329).

The study recruitment was initiated in November 2014. Data collection started in December 2014 and as of submission of this manuscript, 2451 patients from 125 centers from 37 countries have been included. Inclusion of patients will be closed 5 years after initiation of the registry. Quality checks are performed centrally with continuous communication and feedback with the centers to ensure accuracy. First results in terms of descriptive outcomes, patterns of practice, and compliance with the current guidelines are expected soon after patient inclusion is closed. Mid- and long-term outcomes are expected in the first trimester of 2020.

## Discussion

Because prospective data are scarce, many unanswered questions remain about the management and comparative outcomes of UTUC. Conversely, the grade of recommendations is supported by low evidence, although this fact does not always preclude a strong recommendation in a disease with a low prevalence. Besides the rapidly evolving technological field that impacts diagnosis and treatment, several obstacles hinder the implementation of a RCT on the subject [30]. The advanced age of patients, limited availability of armamentarium, high rate of comorbidities of the affected population, and undesired outcomes of standard treatment are some of these obstacles. A recent study showed that comorbidity is inversely associated with being offered participation in clinical oncologic trials even after adjusting for the effects of demographic and socioeconomic factors [36]. Furthermore, it is unlikely that several RCTs may provide recommendations that will fit the whole spectrum of patients and various aspects of the disease. It is under these conditions that outcomes derived from observational cohorts or pragmatic clinical trials will be filling the gaps [37-39]. The biggest clinical challenge in UTUC is the high rate of overtreatment in patients who could be safely offered a kidney-sparing approach as well as the high rate of undertreatment in patients with invasive disease who need more than RNU (ie, [neo]adjuvant systemic therapy to treat occult metastasis).

In line with the definition of a clinical registry, the inclusion criteria of our registry are broad, to capture real-world data on presentation, diagnosis, treatment indication, and outcomes of UTUC. In case of a disease with a low prevalence, registries have a great potential in supporting clinical research and as a source of future trials [29,37]. Registries face practical and operational challenges [29,38,40]. Matters that may compromise the functionality of the CROES-UTUC registry were taken into account at the design phase.

Regarding the logistic organization, the data are centrally collected in a standardized way; they are clinically oriented and adequately frame the affected population. Furthermore, rapid and regular communication and feedback between the central management core of the registry and the participating centers are provided. Regarding quality control, patient data are anonymized and the CROES office monitors the data and sends reminders to encourage the respective principal investigators to ensure completeness and accuracy of essential data elements, especially when missing data are detected. The audit process of the data is independent from the steering committee and participating centers. Secondary data quality checks, cleaning, and analysis will be performed by professional statisticians.

Once these operational questions are addressed with satisfaction, there are still challenges that may hinder the internal validity of a registry as well as the attainment of reliable data [29,37]. Erroneous capture of patients’ information remains a potential source of bias that is difficult to solve in any type of study, especially in studies with retrospective designs. It is our experience that proactive continuous auditing and checking contradictory or exclusive variables minimizes this error, although it is unknown to what extent. Confounding will be considered for any of the planned and proposed studies under clinical and statistical supervision, and information regarding data transfer to the registry (manually or from electronic health records) will be taken into account as possible sources of bias.

Differences exist in local practices and protocols as well as regional variations in standard of care or access to care [37]. Although inherent to any multicentric registry and precisely one of the outcomes to be captured, interpretation may be difficult. Therefore, efforts will be made to adjust for these differences, with a thorough critical analysis. As any center can participate independent of case volume or experience, differences in volume outcomes may surface. These differences may become important and potentially have a positive impact in the quality of care and value-based health care parameters, as highlighted in a recent systematic review [40].

In fact, clinical registries such as pragmatic clinical trials are a valuable complement to RCTs [39] and continue to evolve as a possible niche for RCTs, and incorporation of patient participation becomes a point of reference for evidence-based medicine [40-42]. Despite recognizing the subordinate level of evidence of registry-generated data with respect to RCTs [39], we strongly believe that this internationally prospective collected data will be helpful in understanding the current scenario in management of UTUC and will generate hypotheses to nurture focused RCTs.

With all this in mind, we are aware that the large amount of data collected may be a burden for the centers and that the research output may be limited by missing data. As centers are allowed and encouraged to propose different hypothesis-driven studies, the steering committee together with the statisticians will thoroughly examine the different proposals. The possibility remains that some of the preliminary defined secondary objectives may not be reached, while some others may turn out to be feasible.

In conclusion, the CROES-UTUC registry is a powerful source of information by compiling international clinical data on real-world presentation and treatment of UTUC. The design and logistics of the registry provide adequate operational flow, functionality, and quality control and ensure transparency. An effort is being made to minimize bias in data collection and analysis by means of regular reminders and feedback. The central management and the steering committee guarantee the statistical and clinical support. Lastly, the implication of the centers in proposing secondary objectives and authoring different studies represents, in our view, an additional scientific incentive.

## Appendix

Multimedia Appendix 1 Data management system.

## Abbreviations

CROES: Clinical Research Office of the Endourology Society
DMS: data management system
EAU: European Association of Urology
RCT: randomized controlled trial
RNU: radical nephroureterectomy
URS: ureterorenoscopy
UTUC: upper tract urothelial carcinomas
WHO-ISUP: World Health Organization International Society of Urologic Pathologists
