# Supplementary material for: Evaluation of Patterns of Presentation, Practice, and Outcomes of Upper Tract Urothelial Cancer: Protocol for an Observational, International, Multicenter, Cohort Study by the Clinical Research Office of the Endourology Society
Source: JMIR Res Protoc. 2020 Jan 24;9(1):e15363. doi: 10.2196/15363 (PMC7007587; doi:10.2196/15363)
Supplement: Multimedia Appendix 1 [file resprot_v9i1e15363_app1.docx]

*Data Management System (DMS)*

The data from CROES studies are collected through a web based data collecting and management system. The system can be accessed through the CROES website: [www.croesoffice.org](http://www.croesoffice.org) which makes it convenient to use for participants all over the world and multiple users of the same institution can be connected to the same data.

The DMS consists of several parts, among these the main program, the 'heart' of the system, which enables users to connect and to enter data. Secondly all data entered by the users, login activity and history files are defined in multiple data bases. The DMS contains a program that generates inclusion overview reports and a program that automatically runs queries to check for inconsistencies in the data collected.


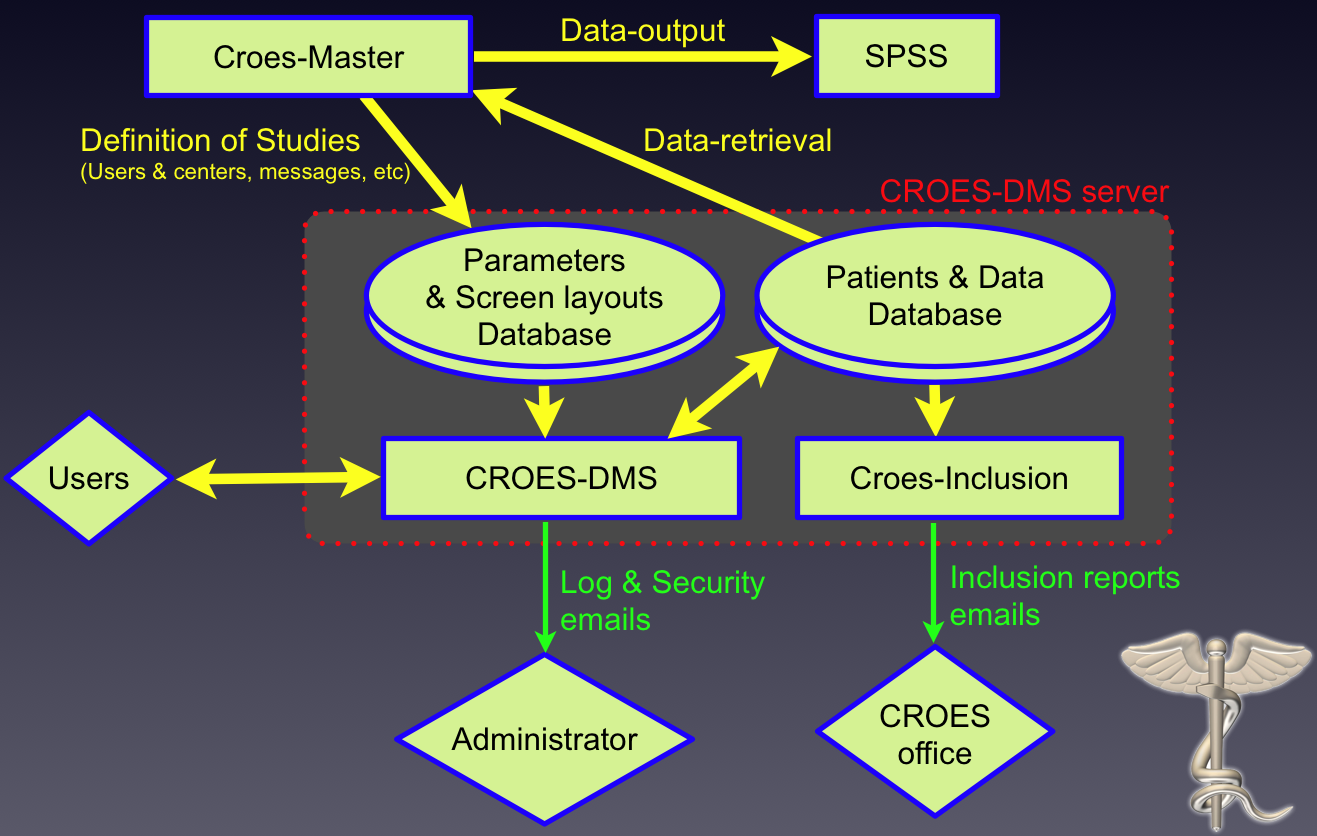


*Security*

It is of great importance for CROES to ensure a high level of security for data collection. To guarantee the safety, the system is located on a secure server in a secure environment with multiple high-speed connections to the Internet. The connection to the system, via htpps, is encrypted for a reliable and secure data transfer.

It is important that your data are stored in a validated system. Validation demonstrates that the system fulfills the user requirements and that, for example, data safety is guaranteed. Based on the results as presented in the validation traceability matrixes document, it was concluded that version 3.0.x of the DMS fulfills all system and user requirements: it functions according to the requested requirements. Furthermore, the system was found to be 21 CRF 11 compliant. A safety analysis, focusing on misuse of the system and/or data, potential data loss, and/or data corruption and nonintended use of the system, was performed and the risk of possible safety issues and potentials hazards were considered to be (very) low.

More information:

The CROES data management system: a glimpse behind the scenes. Van Rees Vellinga S, de la Rosette J. J Endourol. 2011 Jan;25(1):1-5.

AND ITS NAME IS .…. DMS Version 3.0.x. Wijkstra H. J Endourol. 2016 May;30(5):489-492.
